# Exploring Thyroid Function after Kidney Transplantation: The Complex Interplay Unacknowledged in Post-Transplant Care

**DOI:** 10.3390/jcm13123559

**Published:** 2024-06-18

**Authors:** Ita Jelić Pranjić, Lidija Orlić, Ana Carević, Tea Vrdoljak Margeta, Jelena Šimić, Ivan Bubić

**Affiliations:** 1 Department of Nephrology, Dialysis and Kidney Transplantation, University Hospital Center Rijeka, Tome Strižića 3, 51000 Rijeka, Croatia; lidija.orlic@uniri.hr (L.O.); ana.carevic051@gmail.com (A.C.); tvrdoljak88@gmail.com (T.V.M.); jelena.simic@uniri.hr (J.Š.); ivan.bubic@uniri.hr (I.B.); 2 Department of Internal Medicine, The Faculty of Medicine of the University of Rijeka, Braće Branchetta 20, 51000 Rijeka, Croatia; 3 Department of Clinical Sciences I, Faculty of Health Studies, University of Rijeka, Viktora Cara Emina 5, 51000 Rijeka, Croatia

**Keywords:** kidney transplantation, graft function, thyroid function, autoimmune thyroid disease

## Abstract

**Background/Objectives:** The interplay between thyroid function and kidney graft function following kidney transplantation remains incompletely understood. Thyroid disorders are more prevalent in kidney transplant recipients than in the general population and are associated with poorer outcomes. **Methods:** This prospective, single-center study was designed to estimate thyroid function (thyroid-stimulating hormone (TSH), triiodothyronine (T3), free triiodothyronine (FT3), thyroxine (T4), free thyroxine (FT4), as well as anti-thyroid peroxidase antibody (anti-TPO), anti-thyroglobulin antibody (anti-Tg), and thyroid-stimulating immunoglobulin (TSI)) and its influence on kidney graft function among a cohort of 23 kidney transplant recipients during a follow-up period of 12 months. **Results:** Significantly increased levels of T4 and T3 were observed 12 months post-transplantation, with FT3 levels increasing significantly after 6 months. The prevalence of immeasurably low anti-Tg antibodies rose during follow-up. Initially, 8% of patients showed positive TSI, which turned negative for all after 6 months. A statistically significant correlation was found between the initial TSH and the estimated glomerular filtration rate (eGFR) value 6 months after transplantation (*p* = 0.023). The graft function was stable. Proteinuria was statistically significantly lower 12 months after transplantation. **Conclusions:** Identifying additional risk factors, understanding their impact on kidney graft function, and recognizing cardiovascular comorbidities could enhance patient care. Notably, this study marks the first prospective investigation into thyroid function after kidney transplantation in Croatia, contributing valuable insights to the global understanding of this complex interplay.

## 1. Introduction

Chronic kidney disease (CKD) presents a growing public health challenge, affecting approximately 10–15% of the general population [1]. Thyroid dysfunction affects a significant portion of the general population, as well as patients with CKD. Abnormal thyroid function tends to manifest in many CKD patients once their estimated glomerular filtration rate (eGFR) falls below 50% [2]. CKD impacts thyroid function in several ways, including reduced circulating thyroid hormone levels, altered peripheral hormone metabolism, disrupted binding to carrier proteins, potential decrease in thyroid hormone content in tissues, and increased iodine stores in the thyroid glands [3]. Both hypothyroidism and hyperthyroidism adversely affect kidney blood flow, glomerular filtration, tubular function, electrolyte homeostasis, and kidney structure, consequently elevating the risk of cardiovascular diseases (CVDs) [4]. While earlier studies postulated thyroid hormone deficiency as a physiological adaptation among end-stage kidney disease (ESKD) patients [3], contemporary research in both hemodialysis and peritoneal dialysis patients reveals that hypothyroidism, as defined by serum thyroid-stimulating hormone (TSH) levels measured after dialysis initiation, is associated with increased mortality risk, even within the subclinical hypothyroidism TSH ranges [5,6,7]. Among the various patterns of thyroid hormone deficiency observed in CKD, subclinical and overt hypothyroidism, as well as low thyroxine (T4) and low circulating triiodothyronine (T3) levels (referred to as the low-T3 syndrome), are most frequently encountered [8]. In kidney transplant recipients (KTRs), the most common thyroid imbalance is the low-T3 syndrome, with free triiodothyronine (FT3) levels typically falling within the normal range [9]. Autoimmune thyroid disease (AITD), encompassing conditions such as Hashimoto thyroiditis (HT), Graves’ disease (GD), and self-atrophic thyroiditis, stands as the leading cause of both hyperthyroidism and hypothyroidism [10]. Among these, HT and GD are especially common in clinical settings and can cause secondary glomerular diseases through various mechanisms [11,12].

Kidney transplantation is the method of choice in the treatment of patients with ESKD. Despite the normalization of eGFR post-transplantation, functional thyroid gland disorders remain more prevalent in KTRs compared to the general population, and they are correlated with adverse cardiovascular outcomes. Persistent low-T3 syndrome has been observed following kidney transplantation, and a low pre-transplantation T3 level is linked to poorer graft survival. Immunosuppressive therapy plays a significant role in these outcomes [13]. Furthermore, ESKD patients diagnosed with HT who undergo kidney transplantation face a significantly heightened risk of transplant failure compared to KTRs without an HT diagnosis [14].

As of now, there remains a scarcity of prospective studies delving into the relationship between thyroid function following kidney transplantation and its impact on kidney graft function, leaving the interplay between the two incompletely understood.

## 2. Materials and Methods

This prospective, single-center study was designed to estimate thyroid function among a contemporary cohort of KTRs during a follow-up period of 12 months. The study included 23 patients who received kidney transplantation between January 2021 and December 2022. Exclusion criteria were age of <18 years, previous thyroid disorders, malignant diseases, acute systemic or infectious diseases. Laboratory parameters were measured immediately after kidney transplantation and then 6 and 12 months after transplantation. KTRs were administered a standard three-drug immunosuppressive regimen consisting of glucocorticoids, calcineurin inhibitors, and mycophenolate mofetil. Induction therapy was administered using either anti-interleukin-2 receptor antibodies or antithymocyte globulin, tailored based on donor and recipient characteristics.

A complete assessment of thyroid function according to serum levels of thyroid-stimulating hormone (TSH), triiodothyronine (T3), free triiodothyronine (FT3), thyroxine (T4), and free thyroxine (FT4) (Atellica Solution (Siemens, Erlangen, Germany)), as well as measurement of the thyroid antibodies thyroid-stimulating immunoglobulin (TSI, Immulite 2000XPi (Siemens, Erlangen, Germany)), anti-thyroidperoxidase antibody (anti-TPO, Atellica Solution (Siemens, Erlangen, Germany)), and anti-thyroglobulin antibody (anti-Tg, Atellica Solution (Siemens, Erlangen, Germany)). In addition to the above, we analyzed the function of the kidney graft by determining urea and creatinine values (Roche diagnostics, Mannheim, Germany) and calculating estimated glomerular filtration rate (eGFR) using the CKD EPI equation. Other measured laboratory findings included standard routine laboratory tests: hemoglobin (Beckman Coulter DxH800 (Beckman Coulter, Brea, CA, USA)), glucose, calcium, phosphorus, albumin, cholesterol, triglycerides, proteinuria (Roche Cobas 6000 c501 (Roche diagnostics, Mannheim, Germany)), low-density lipoprotein (LDL, calculated using Friedwald equation), high-density lipoprotein (HDL, Beckman Coulter AU480 (Beckman Coulter, Brea, CA, USA), and parathyroid hormone (PTH, Roche Cobas 6000 e601 (Roche diagnostics, Mannheim, Germany)).

## 3. Statistical Analysis

Statistical analysis was performed using MedCalc^®^ Statistical Software version 22.009 (MedCalc Software Ltd., Ostend, Belgium; https://www.medcalc.org, accessed on 29 May 2024; 2023). Based on data from a previously published study [15], the number of participants required for this study was determined. According to the analysis conducted, at least 15 participants needed to be included in the study as the conclusions were made with a Type I error of 5% (*p* < 0.05) to achieve a study power of 90% (β = 0.1).

The quantitative variables are presented as mean and standard deviation or as median and interquartile range depending on the distribution of the data (normal or non-normal distribution). Age, regardless of distribution, is shown by median and range (minimum–maximum). Categorical variables are presented as a proportion. Difference between the tested parameters at the beginning of the study and after 6 months was tested using the Wilcoxon test for quantitative variables and chi-square for qualitative variables and the difference between the three points, immediately after, 6 months after, and 12 months after kidney transplantation was tested by the Friedman test for quantitative variables and the chi-square for qualitative variables. Correlation analysis (Spearman’s correlation coefficient) was used to determine the connection (correlation) between variables. Statistically significant association (with *p* < 0.05) was interpreted according to Colton’s criterion (for r from 0 to 0.25 or from 0 to −0.25 as absence of association; for r from 0.26 to 0.50 or −0.26 to −0.50 as weak association; for r from 0.51 to 0.75 or −0.51 to −0.75 as moderate to good association; for r from 0.76 to 1 or −0.76 to −1 as very good to excellent association).

To demonstrate the impact of changes in thyroid status on kidney function in KTRs, we used a scatter plot and a regression equation. This enabled us to display the relationship between variations in TSH and thyroid hormone levels and changes in eGFR and creatinine levels, comparing results 12 months post-transplantation to those immediately after transplantation.

## 4. Results

The demographic and clinical characteristics of patients are reported in Table 1.

The laboratory results are shown in Table 2 and the results for thyroid hormones and antibodies are shown in Table 3.

Table 2 and Table 3 also show the significance levels (*p* values) for each determined parameter. The P1 value represents the level of significance of the difference between the centers of a given parameter for all patients between the beginning of the study and after 6 months and the P2 value after 12 months.

During the follow-up period, kidney graft function was stable and there was no statistically significant difference in creatinine and eGFR values (Table 2). Proteinuria was statistically significantly lower 12 months after transplantation compared to the beginning of the study and 6 months after. A statistically significantly higher hemoglobin concentration was found in patients after 6 and 12 months compared to the beginning of the study. Calcium concentration increased statistically significantly 6 months after transplantation compared to the beginning of the study, while PTH concentration decreased statistically significantly 12 months after transplantation compared to the beginning of the study and 6 months after. Albumin concentration increased statistically significantly 6 months after transplantation. TSH concentration was within the reference intervals during the entire follow-up period. The concentration of T4 and T3 significantly increased 12 months after transplantation, while the concentration of FT3 increased significantly at 6 months after transplantation (Table 3). Immediately after transplantation, 50% of patients had immeasurably low anti-TPO antibodies, and 86% had immeasurably low anti-Tg antibodies. The proportion of patients with immeasurably low anti-TPO antibodies was significantly lower 6 months after transplantation, and the proportion increased again after 12 months; however, it was still significantly lower compared to the beginning of the study. On the other hand, the proportion of patients with immeasurably low anti-Tg antibodies increased during follow-up. Also, 8% of patients had a positive TSI immediately after transplantation, and 6 months after transplantation all patients had a negative TSI.

**Figure 1:**
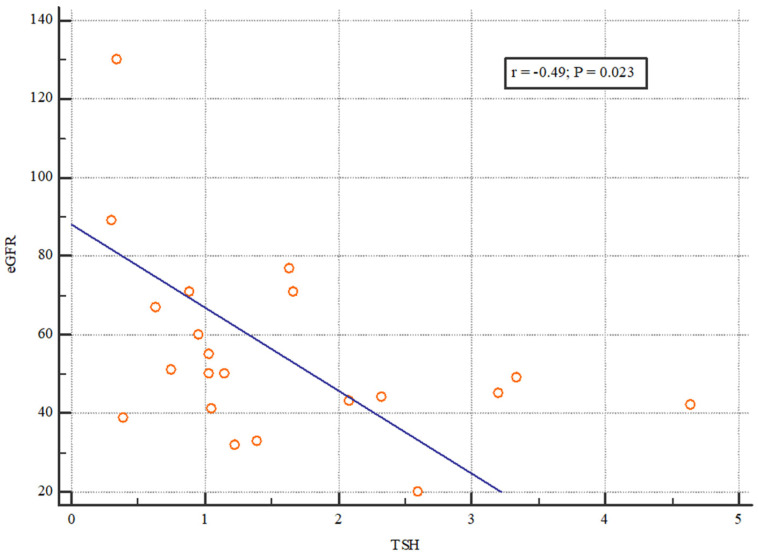
The correlation of eGFR and TSH 6 months after kidney transplantation. The figure shows a statistically significant correlation between the initial TSH and the eGFR value 6 months after transplantation (*p* = 0.023). The Spearman’s correlation coefficient was −0.493, which indicates a weak correlation between the two parameters.

To examine the relationship between the initial TSH and the function of the graft throughout the follow-up period, the correlation between the initial TSH and the creatinine concentration, as well as eGFR after 6 and 12 months, was tested. A statistically significant correlation was found between the initial TSH and the eGFR value 6 months after transplantation (*p* = 0.023). The Spearman’s correlation coefficient was −0.493, which indicates a weak correlation between the two parameters (Figure 1).

**Figure 2:**
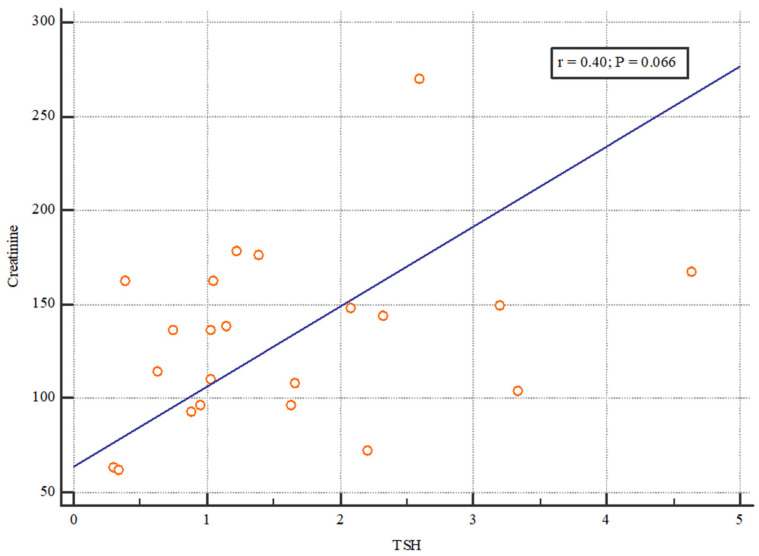
The correlation between TSH and creatinine 6 months after kidney transplantation. The figure shows no correlation between the initial TSH and creatinine concentration 6 months after transplantation.

On the other hand, there was no correlation between the initial TSH and creatinine concentration 6 months after transplantation (Figure 2).

**Figure 3:**
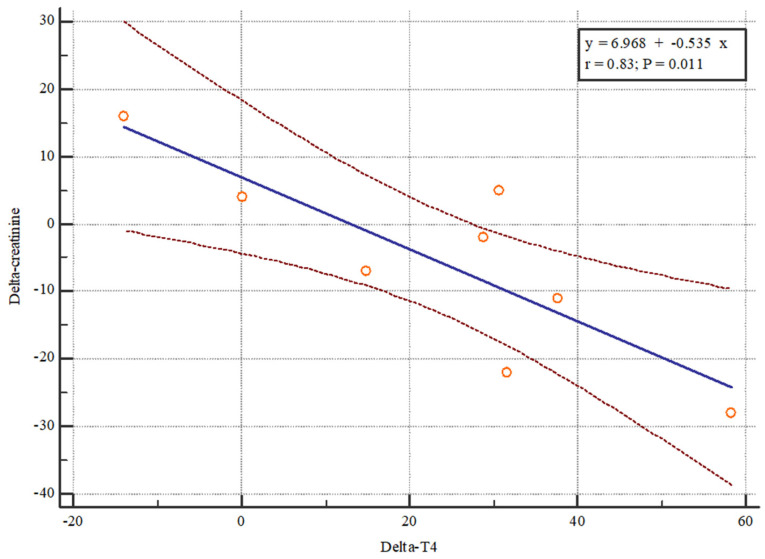
Scatter diagram (with regression line and 95% confidence interval) of delta creatinine (creatinine^24months^-creatinine^0^) and T4 (T4^24months^-T^0^) 12 months after kidney transplantation. The scatter diagram shows how changes in total T4 levels affect changes in creatinine levels, an increase in total T4 levels leads to a decrease in creatinine levels.

To examine the impact of thyroid status on kidney function after transplantation, we calculated the changes in TSH and thyroid hormone levels (levels at 12 months—levels immediately after transplantation) and correlated them with changes in eGFR and creatinine levels (levels at 12 months—levels immediately after transplantation). The analysis showed that changes in total T4 levels affect changes in creatinine levels, specifically, an increase in total T4 levels leads to a decrease in creatinine levels (Figure 3).

**Figure 4:**
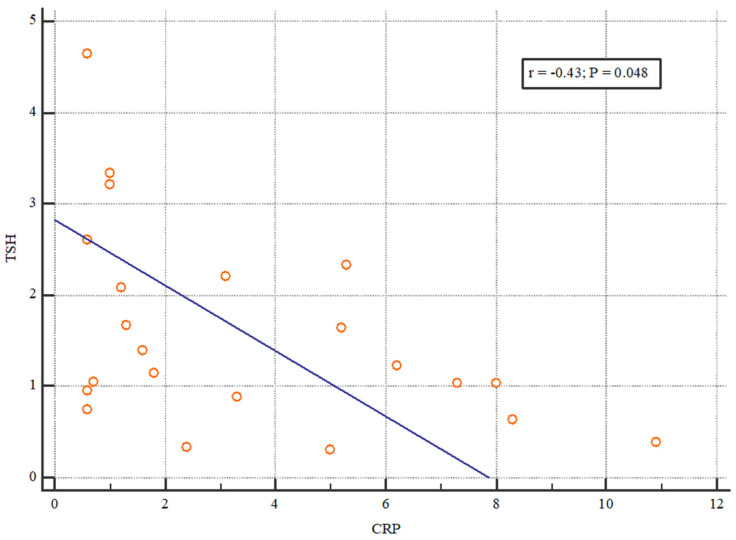
The correlation between CRP and TSH values after transplantation. The Spearman’s correlation coefficient was −0.43 which shows a weak negative correlation between TSH and CRP.

**Figure 5:**
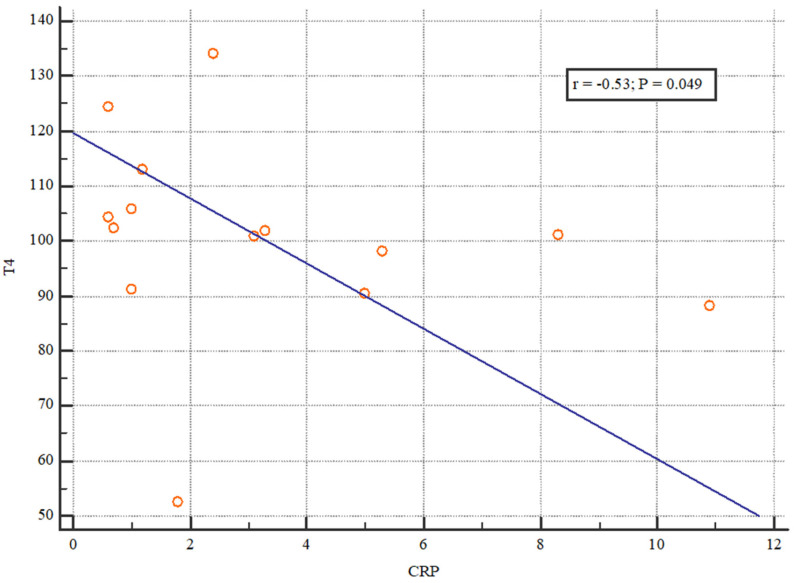
The correlation between CRP and T4 after transplantation. The Spearman’s correlation coefficient was −0.53 which shows a moderate negative correlation between T4 and CRP.

We evaluated the relationship between thyroid status and CRP as an inflammation parameter. Our analysis of initial CRP values and thyroid hormones revealed a weak negative correlation between TSH and CRP and a moderate negative correlation between T4 and CRP (Figure 4 and Figure 5).

We performed regression analyses to determine if changes in CRP levels influenced changes in thyroid hormones and found no significant associations.

The amount of hospitalizations/number of patients was 1.0 after 6 months and 1.3 after 12 months. The duration of hospitalizations during the follow-up period was 2 (0–8.5) days after 6 months and 3 (0–17) days after 12 months. It is evident that the frequency of hospitalizations per patient increased over time, as did the duration of hospitalizations. The most common cause of hospitalizations was worsening of kidney graft function and urinary infection. During the duration of the study, one death was recorded after 12 months (out of 18 patients who were monitored for 12 months), which makes for a survival rate of 100% for 6 months and 94.4% for 12 months.

## 5. Discussion

The serum concentration of serum TSH typically remains within normal limits in CKD. However, the TSH response to exogenous thyrotropin-releasing hormone (TRH) often exhibits a blunted and delayed pattern, with an extended time required to return to baseline levels. This delayed recovery may stem from reduced kidney clearance, as both TSH and TRH are normally cleared by the kidneys. Nonetheless, the blunted hormone response also indicates potential dysfunction at the hypothalamic–pituitary level, which could be induced by uremic toxins. TSH release appropriately adjusts to changes in circulating thyroid hormone levels. Exogenous administration of T3 decreases TSH levels and can fully suppress the secretory response to exogenous TRH. Notably, TSH production increases appropriately in response to thyroid ablation. This latter response holds clinical significance, particularly since TSH levels should rise in uremic patients developing hypothyroidism [16].

The most prevalent thyroid function abnormality observed in CKD patients is a decrease in T3 levels, particularly total T3 compared to FT3. Termed the “low-T3 syndrome”, this phenomenon in CKD arises from various factors. Chronic metabolic acidosis and protein malnutrition impact iodothyronine deiodination and T3 protein binding, thereby reducing the peripheral conversion of T4 to T3 and its protein binding. Moreover, impaired renal iodine handling elevates serum iodine levels, leading to a prolonged Wolff–Chaikoff effect [17]. Low T3 levels in CKD patients correlate with elevated markers of inflammation, malnutrition, heightened endothelial dysfunction, poorer cardiac function, reduced survival rates, and increased all-cause and cardiovascular mortality in certain studies [18]. T4 levels are typically lower in many CKD patients, but FT4 levels may vary from low to normal due to impaired T4 protein binding in CKD. These mechanisms likely reflect the body’s physiological adaptations to CKD, aimed at reducing protein nitrogen turnover, protein catabolism, and nitrogenous waste load. Notably, there is no increased incidence of AITD in CKD patients, although it may coexist with other autoimmune conditions linked to CKD, such as lupus nephritis or type 1 diabetes mellitus [19].

KTRs, by virtue of their CKD status, exhibit distinct characteristics compared to non-transplant CKD patients. Long-term immunosuppressive and corticosteroid therapy, which impact various organ systems, including the thyroid gland, contribute to these differences. Changes in thyroid function consequentially affect kidney graft function. A retrospective study by Schairer et al. involving 398 euthyroid kidney transplant recipients revealed that an increase in TSH levels over 12 months during the second post-transplant year correlated with an annual decline in eGFR of 1.34 mL/min per unit (μIU/mL) TSH. This finding underscores the significance of individual changes in TSH values over time in influencing eGFR, independent of age, gender, body mass index, and thyroid hormone replacement therapy [20]. We also found a correlation between the two mentioned parameters within the six months after transplantation. Furthermore, a correlation between FT3 levels and serum creatinine/eGFR suggests that FT3 levels correlate with long-term kidney graft function [21]. Low FT3 concentrations, present in up to half of KTRs in some studies [22], are independently associated with endothelial damage and immunosuppressive treatment [23]. Our study showed a statistically significant rise in the levels of FT3 within 6 months after transplantation. The pronounced reduction in FT4 and FT3 levels observed in KTRs experiencing delayed graft function (DGF) compared to those with normal graft function is regarded as an outcome rather than a primary cause of DGF. This phenomenon aligns with the concept of “euthyroid sick syndrome”, attributable to elevated doses of methylprednisolone, heightened severity of reperfusion injury, inflammation, and prolonged uremic state [24]. Rotondi et al. found that KTRs exhibiting pre-transplantation FT3 levels < 3.1 pmol/L are linked to lower five-year death-censored graft survival rates [25], indicating a potential prognostic value for assessing graft failure risk [26]. Notably, hyperthyroidism occurrence post-transplantation due to immunosuppressive therapy is exceedingly rare [27]. Our results suggest a plausible influence of immunosuppression on antibody levels, potentially explaining the rare incidence of AITD following kidney transplantation. Kidney transplantation is the method of choice in the treatment of patients with ESKD. Graft function is influenced by various factors, including thyroid function, as evidenced by the previous literature. Maintenance immunosuppression post-transplantation also impacts thyroid function.

## 6. Conclusions

This is the first prospective study of thyroid function after kidney transplantation in Croatia and one of the few in the world. Our results showed a statistically significant increase in FT3 and T4 values, which is, considering the results of earlier studies, beneficial for our KTRs’ graft function since it is suggested that FT3 levels correlate with the kidney graft function in the long term. We also found a negative correlation between TSH and eGFR within the six months after transplantation.

In conclusion, the intricate interplay between thyroid and kidney graft function underscores the critical importance of evaluating thyroid function in transplant recipients. Both organs, the thyroid and the kidney, exert profound influences on each other’s functionality, and disruptions in one can significantly impact the other. As evidenced by emerging research, thyroid dysfunction in kidney transplant recipients can lead to a myriad of complications, ranging from CVD to metabolic disturbances, thereby jeopardizing the overall success of kidney transplantation. Hence, thorough and routine assessment of thyroid function should be integrated into the post-transplant care. By doing so, healthcare providers can optimize patient outcomes, enhance long-term graft survival, and improve the quality of life for KTRs. This approach to KTR underscores the imperative of comprehensive management strategies in transplant medicine.

The limitations of our study include a relatively small number of patients and further studies to clarify the connection of thyroid function and kidney graft function after kidney transplantation are needed.

## Figures and Tables

**Table 1:** Demographic and clinical characteristics of patients.

Age (Years)	53 (16–75) *
Sex (male/female)	17/6 **
Body mass index (kg/m^2^)	25.1 (21.7–28.7) ***
Dialysis modality before Tx, N (%)	
Hemodialysis (HD)	14 (63.7%)
Peritoneal dialysis (PD)	5 (22.7%)
Bimodal dialysis (HD + PD)	3 (13.6%)
Dialysis duration before Tx (years)	3 (2.0–4.8) ***
Primary kidney disease, N (%)	
Nephroangiosclerosis	4 (17.4%)
Polycystic kidney disease	3 (13%)
IgA nephropathy	5 (21.7%)
Obstructive nephropathy	1 (4.4%)
Other	10 (43.5%)
Comorbidities, N (%)	
Arterial hypertension	21 (91.3%)
Ischemic heart disease	2 (6%)
Diabetes mellitus	3 (13%)
Kidney donor	
Age, years	51 (26–65) *
Male/Female (%)	58/42
Immunosuppression, N (%)	
Induction	23 (100%)
Mycophenolate mophetil	22 (95.6%)
Tacrolimus	23 (100%)
Glucocorticoids	23 (100%)

* median and range; ** relative share; *** median and interquartile range.

**Table 2:** Laboratory findings during the follow-up period.

	Immediately after Tx	6 Months after Tx	12 Months after Tx	P1*	P2**
Red blood cells (×10^12^/L)	3.40 ± 0.55	4.58 ± 0.50	4.69 ± 0.45	<0.001	<0.001
Hemoglobin (g/L)	103.4 ± 8.1	131.1 ± 14	133.4 ± 14.2	<0.001	<0.001
CRP (mg/L)	3.4 ± 2.9	6.7 ± 13.4	3.9 ± 5.3	0.680	0.862
Glucose (mmol/L)	5.2 ± 1.2	6.2 ± 2.0	5.6 ± 1.4	0.088	0.256
Creatinine (µmol/L)	127.1 ± 52.5	132.4 ± 46.3	140.3 ± 45.5	0.145	0.514
eGFR (mL/min/1.73 m^2^)	60.0 ± 24.1	54.7 ± 22.7	51.6 ± 18.4	0.088	0.497
Calcium (mmol/L)	2.3 ± 0.2	2.5 ± 0.1	2.6 ± 0.1	<0.001	<0.001
Phosphorus (mmol/L)	1.0 ± 0.6	0.88 ± 0.2	0.9 ± 0.2	0.840	0.507
PTH (pmol/L)	24.8 ± 16.4	16.2 ± 11.5	12.4 ± 7.1	0.129	0.032
Cholesterol (mmol/L)	5.1 ± 1.1	5.2 ± 0.9	4.9 ± 0.8	0.186	0.896
HDL (mmol/L)	1.3 ± 0.3	1.5 ± 0.6	1.4 ± 0.6	0.050	0.851
LDL (mmol/L)	2.5 ± 0.8	2.8 ± 0.8	2.7 ± 0.6	0.296	0.521
Triglycerides (mmol/L)	0.8 ± 0.2	2.0 ± 0.9	2.0 ± 0.9	0.384	0.497
Albumin (g/L)	38.6 ± 4.0	44.0 ± 4.4	45.6 ± 2.8	<0.001	<0.001
Proteinuria (mg/L)	473.8 ± 889.8	167.9 ± 173.8	130.5 ± 115.9	0.110	0.018
Tacrolimus (ng/mL)	7.6 ± 2.9	7.8 ± 2.3	7.6 ± 2.8	0.948	0.660

All variables are presented as mean and standard deviation. P1*—significance level between the beginning of the study and after 6 months (N = 23); P2**—significance level between the beginning of the study and after 6 and 12 months (N = 18). Tx, kidney transplantation; CRP, C reactive protein; eGFR, estimated glomerular filtration rate; PTH, parathyroid hormone; HDL, high-density lipoprotein; LDL, low-density lipoprotein.

**Table 3:** Thyroid hormone and antibody levels during the follow-up period.

	Immediately after Tx	6 Months after Tx	12 Months after Tx	P1*	P2**
TSH (mIU/L)	1.186 (0.884–2.207)	1.508 (1.060–2.401)	1.901 (0.976–2.312)	0.482	0.651
T4 (nmol/L)	101.4 (91.1–105.9)	113.0 (98.4–124.5)	128.01 (88.4–138.6)	0.169	<0.001
FT4 (pmol/L)	15.6 (14.0–16.9)	15.8 (13.9–17.6)	15.3 (14.0–19.6)	0.632	0.926
T3 (nmol/L)	1.5 (1.4–1.7)	1.6 (1.4–1.9)	1.8 (1.5–2.1)	0.169	0.070
FT3 (pmol/L)	4.6 (4.4–5.0)	5.1 (4.6–5.4)	5.4 (4.6–5.8)	0.004	0.085
Anti-TPO (immeasurable/neg %) ^#^	50/50	18/82	19/81	<0.001	<0.001
Anti-Tg (immeasurable/neg %) ^#^	86/14	94/6	94/6	0.006	0.068
TSI (neg/pos%) ^#^	92/8	100/0	100/0	0.004	<0.001

All variables are presented as median and interquartile range, except for #, which are presented as percentages. P1*—significance level between the beginning of the study and after 6 months (N = 23); P2**—significance level between the beginning of the study and after 6 and 12 months (N = 18). Tx, kidney transplantation; TSH, thyroid-stimulating hormone; T4, thyroxine; FT4, free thyroxine; T3, triiodothyronine; FT3, free triiodothyronine; Anti TPO, anti-thyroidperoxidase antibody; Anti-Tg, anti-thyroglobulin antibody; TSI, thyroid-stimulating immunoglobulin.

## Data Availability

The data presented in this study are available on request from the corresponding author. Due to privacy and ethical restrictions, the data contain personal patient information, including names, and therefore are not publicly available.
